# Prognosis following emergency surgery for ulcerative colitis in elderly patients

**DOI:** 10.1007/s00595-013-0563-z

**Published:** 2013-04-04

**Authors:** Hiroki Ikeuchi, Motoi Uchino, Hiroki Matsuoka, Toshihiro Bando, Akihiro Hirata, Yoshio Takesue, Naohiro Tomita, Takayuki Matsumoto

**Affiliations:** 1 Inflammatory Bowel Disease Center, Hyogo College of Medicine, 1-1 Mukogawa-cho, Nishinomiya, Hyogo 663-8501 Japan; 2 Department of Infection Control and Prevention, Hyogo College of Medicine, 1-1 Mukogawa-cho, Nishinomiya, Hyogo 663-8501 Japan; 3 Department of Surgery, Hyogo College of Medicine, 1-1 Mukogawa-cho, Nishinomiya, Hyogo 663-8501 Japan

**Keywords:** Ulcerative colitis, Elderly patients, Emergency operation, Prognosis

## Abstract

**Purpose:**

Since 2000, the incidence of ulcerative colitis (UC) in patients over 60 years old has been rapidly increasing. We reviewed our surgical experience of elderly patients with UC treated at our hospital.

**Methods:**

Patients aged 60 years or older at the time of surgery were defined as “elderly”. The medical records of all elderly patients who underwent surgery for UC during a 26-year period were retrospectively analyzed.

**Results:**

The prognosis of elderly patients who underwent emergency surgery was extremely poor: 8 (26.7 %) of 30 such patients died within 30 postoperative days (PODs), whereas only 1 (0.88 %) of 114 who underwent elective surgery died within 30 PODs. Respiratory tract infection and sepsis resulting from methicillin-resistant *Staphylococcus aureus* or mycotic infection were the most common causes of death after emergency surgery.

**Conclusion:**

The prognosis of elderly UC patients undergoing emergency surgery is very poor; thus, physicians and surgeons should collaborate to treat severe and fulminant disease, to optimize the timing of surgery. Early decisions about emergency surgery for UC will reduce postoperative mortality, especially in elderly patients.

## Introduction

With the aging population, recognizing ulcerative colitis (UC) in older patients is becoming important. However, it is unclear whether the high mortality of elderly patients with UC, reaching 19 % in some reports, is due to the disease process itself or to the adverse effects of concomitant illnesses [1–5].

Approximately, 10 % of UC patients undergo colectomy within 10 years of its diagnosis [6]. However, surgery is usually performed for UC only if a patient does not respond to medical therapy, or if an acute complication of UC (such as toxic megacolon), or colonic dysplasia or cancer, develop. Total colectomy with an ileal pouch anal anastomosis (IPAA) is the preferred procedure, although permanent ileostomy without an ileal pouch may be more appropriate for elderly patients.

We reviewed our surgical experience of elderly patients suffering from UC, who were treated at our hospital. We analyzed the differences between elective and emergency UC surgery in patients aged 60 years or older, in relation to patient characteristics, indications for surgery, surgical procedures, and short-term outcomes.

## Patients and methods

### Patients

We identified 1275 patients with UC, who underwent colectomy at Hyogo College of Medicine between August 1, 1984 and December 31, 2010. In this study, ‘elderly’ was defined as 60 years of age or older. The medical records of all elderly patients who underwent surgery for UC during that 26-year period (*n* = 144) were retrospectively analyzed. Patients were divided into elective and emergency surgery groups. The diagnosis of UC was based on recognized radiological, endoscopic, and histopathological criteria.

### Definition

Mortality was defined as death within 30 days of, or directly related to, the surgical procedure. Surgery was defined as ‘elective’ if the decision to operate for UC was made prior to admission to the hospital, whereas the decision to perform ‘emergency’ colectomy was decided during or after admission for acute complications or for UC refractory to in-hospital intensive medical management.

### Statistical analysis

Grouped data are presented as the median and range. The results were compared using Mann–Whitney *U* and χ^2^ tests, with probability values less than 0.05 considered significant.

## Results

### Age at the time of surgery

**Fig. 1:**
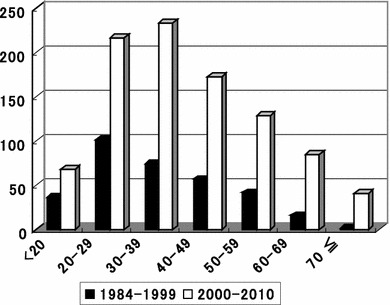
Changes over time in age distribution at the time of surgery. During the early period, the median age at the time of surgery was in the 20s, whereas that during the later period had shifted to the 30s. The proportion of patients aged over 60 years old has also been increasing

Figure 1 shows the changes over time in the distribution of age at the time of surgery. From 1984 to 1999, the highest incidence of surgery was in patients 20–29 years old, whereas from 2000 to 2010 the highest incidence was in those aged 30–39 years. Of 330 patients who underwent surgery in the early period, 18 (5.5 %) were elderly, whereas of 945 patients who underwent surgery in the latter period, 126 (13.3 %) were elderly. This difference between the study periods was significant.

### Patient backgrounds and preoperative medical treatments

Table 1 summarizes the characteristics of the 144 elderly patients. There were 30 elderly patients in the emergency surgery group and 114 in the elective surgery group. Sex and age at surgery were not significantly different between the groups, although the duration of disease in the emergency surgery group was significantly shorter. The incidence of severe or fulminant type UC was significantly greater in the emergency surgery group.

**Table 1:** Patient characteristics and preoperative medical treatments

	Emergency surgery (*n* = 30)	Elective surgery (*n* = 114)	*P*
Age in years at time of surgery (range)	66 (60–81)	66 (60–85)	0.56
Sex, M/F	19/11	69/45	0.89
Duration of disease in months (range)	21 (1–225)	83 (2–409)	<0.01
Pancolitis (%)	27 (90 %)	78 (68 %)	0.37
Severe or fulminant type (%)	25 (83 %)	12 (11 %)	<0.01
Steroid total dose in mg (range)	5,850 (0–1,10,000)	9,000 (0–75,000)	0.79
Steroid daily dose in mg (range)	50 (0–80)	10 (0–60)	<0.01
Immunosuppressants, no. of patients (%)	5 (17 %)	18 (16 %)	0.92
LRT, no. of patients (%)	10 (33 %)	44 (39 %)	0.72

*LRT* leukocyte removal therapy

The daily dose of steroids preoperatively was significantly higher in the emergency surgery group than in the elective surgery group. In contrast, the daily dose of steroids in patients who died (50 mg, range 30–60 mg) did not differ significantly from that of those who survived for at least 30 days after emergency surgery (55 mg, 0–80 mg) (*P* = 0.83). Moreover, the rates of immunosuppressant and leukocyte removal therapy (LRT) use did not differ significantly between the groups.

### Surgical indications and postoperative mortality

Table 2 shows the surgical indications and number of postoperative deaths. Massive hemorrhage was the most common indication for surgery in the emergency surgery group. In the elective surgery group, 83 (72.8 %) received refractory medical treatment and 31 (27.2 %) underwent surgery for colitis-associated colorectal cancer or dysplasia. The preoperative indications associated with the worst postoperative mortality were severe or fulminant type (*n* = 7) UC, as 3 (42.9 %) of those patients died within 30 days of surgery.

**Table 2:** Surgical indications and mortality

Surgical indication	No. (%)	No. of deaths (%)
Emergency surgery (*n* = 30)		
Massive hemorrhage	10 (6.9)	4 (40.0)
Toxic megacolon	8 (5.6)	0 (0)
Severe or fulminant type	7 (4.9)	3 (42.9)
Free perforation	5 (3.5)	1 (20.0)
Elective surgery (*n* = 114)		
Refractory to medical therapy	83 (57.6)	1 (1.2)
Cancer/dysplasia	31 (21.5)	0 (0)

### Age at surgery and mortality

Table 3 shows the age at surgery and the rates of mortality. The prognosis of elderly patients who underwent emergency surgery was extremely poor. Eight (26.7 %) of 30 elderly patients died within 30 days of surgery, whereas only 1 (0.88 %) of 114 patients who underwent elective surgery died during that early postoperative period.

**Table 3:** Age at time of surgery and mortality

	Emergency surgery	Elective surgery	*P*
Under 60 years old (*n* = 1131)	6/231 (2.6 %)	7/900 (0.78 %)	0.02
60 years old and older (*n* = 144)	8/30 (26.7 %)	1/114 (0.88 %)	<0.01

### Causes of death

Table 4 shows the causes of death of the elderly patients. Of the 8 patients in the emergency surgery group who died during the early postoperative period, the cause of death was pneumonia in 4, intra-abdominal sepsis in 3, and perforation of the colitis associated with transverse colon cancer in 1. Respiratory complications and sepsis derived from methicillin-resistant *Staphylococcus aureus* (MRSA) or fungal infection were frequent causes of death. Three of the four patients in the emergency surgery group who died of pneumonia had MRSA and fungal infections, while the other patient died of pneumonia with a *Pseudomonas aeruginosa* infection. The single patient from the elective surgery group, who died during the early postoperative period, died of intra-abdominal sepsis caused by leakage from the stump following a Hartmann procedure.

**Table 4:** Causes of death of elderly patients

Cause of death	
Emergency surgery (8/30, 26.7 %)	
Pneumonia	4
Sepsis	3
Perforation (cancer)	1
Elective surgery (1/114, 0.88 %)	
Sepsis	1

### Initial operative procedure and postoperative mortality

Table 5 shows the initial operative procedures and postoperative mortality. Five (26.3 %) of 19 patients who underwent emergency total colectomy with a mucous fistula or Hartmann procedure died within 30 days of their operation.

**Table 5:** Initial operative procedures and postoperative mortality

Initial operative procedure	60–69 years (*n* = 101)				70 years and older (*n* = 43)			
	Emergency surgery (*n* = 24)		Elective surgery (*n* = 77)		Emergency surgery (*n* = 6)		Elective surgery (*n* = 37)	
	No. of deaths		No. of deaths		No. of deaths		No. of deaths	
TC	14	4	4	0	5	1	7	1
IPAA (hand sewn) with ileostomy	7	2	48	0	0	0	0	0
IPAA (hand sewn) without ileostomy	0	0	5	0	0	0	0	0
IPAA (stapled anastomosis)	1	0	6	0	0	0	1	0
IRA	0	0	1	0	0	0	3	0
TPC	1	0	13	0	1	0	26	0
Miscellaneous	1	1	0	0	0	0	0	0
Total	24	7 (29.2 %)	77	0 (0 %)	6	1 (16.7 %)	37	1 (2.7 %)

*TP* total colectomy with mucous fistula or Hartmann procedure, *IPAA* ileal pouch anal anastomosis, *IRA* ileorectal anastomosis, *TPC* total proctocolectomy

### Final operative procedures

Table 6 lists the final operative procedures performed for our elderly patients. Of 101 patients whose age at the time of surgery was 60–69 years old, 84 (83.2 %) underwent sphincter-preserving surgery, whereas only 9.3 % of those aged 70 years old or older at the time of surgery underwent that type of surgery.

**Table 6:** Final operative procedures in elderly patients

	60–69 years (*n* = 101)	70 years and older (*n* = 43)
IPAA (hand sewn anastomosis)	72	0
IPAA (stapled anastomosis)	11	1
IRA	1	3
Sphincter-preserving operation (%)	84 (83.2)	4 (9.3)
Total proctocolectomy with end ileostomy	16	35
Total colectomy with end ileostomy	0	4
Miscellaneous	1	0

*IPAA* ileal pouch anal anastomosis, *IRA* ileorectal anastomosis

## Discussion

Elderly patients represent an increasing proportion of the population with UC. Restorative proctocolectomy with an IPAA continues to be the surgical technique of choice for UC; however, the results of that procedure tend to be worse in older patients, although the procedure is associated with reasonably good outcomes overall, in terms of efficacy and morbidity. In patients older than 65 years, the major concern of this procedure relates to an increased frequency of diurnal incontinence and nocturnal leakage [7]. Thus, age alone is not considered a contraindication to IPAA.

In the present study, 83.2 % of the patients aged 60–69 years vs. 9.3 % of those aged 70 years and older underwent a sphincter-preserving operation. When considering this type of surgery, informed consent must be obtained after consultation between the patient and attending physician.

There is some debate regarding disease activity in elderly UC patients. Lakatos et al. reported that the disease course of UC was milder in elderly patients, with fewer fulminant episodes and lower systemic steroid exposure. However, although the absolute risk was low, UC-associated dysplasia and/or cancer developed sooner in their elderly patients [8]. In contrast, Beltrán reported that the disease course of UC was generally more severe in elderly patients, and the mortality of hospitalized patients with both UC and CD was estimated to be three to five times higher than that of patients younger than 65 years [9]. Although older patients were thought to have higher rates of complications, while the mortality rates of patients requiring urgent surgery have been reported at as high as 50 % [10–12], the number of elderly patients in those studies was small. Moreover, Almogy et al. [13] reported emergency surgery, a low level of albumin, and male sex as predictors of an adverse outcome in their elderly UC population. Fewer complications and a lower death rate should be expected in elderly UC patients undergoing elective procedures.

The medical options for treating UC have been increasing. In Japan, the early and aggressive administration of immunosuppressants was systematically implemented in the early 2000s, while biological treatments only became available in 2010. In the Stockholm cohort study, giving infliximab to patients older than 60 years old was associated with an increased risk of serious side effects and higher mortality [14].

The prognosis after emergency surgery for UC is extremely poor for elderly patients; thus, it remains controversial as to whether they should be treated with the same therapy as younger patients. Ananthakrishnan et al. [15] reported a significant difference in mortality between patients who underwent surgery and those who did not. This is possibly because medical salvage therapies, including immunomodulatory and/or biological therapies, may increase morbidity and mortality, suggesting that early surgery is associated with lower mortality in older patients. Moreover, de Silva et al. [16] reported that patients who were admitted to hospital under emergency conditions and did not respond to medical treatment had a worse outcome when surgery was performed 14 or more days after their presentation. Since the prognosis associated with emergency surgery under these conditions is very poor, physicians and surgeons should collaborate to treat severe and fulminant UC, so as to avoid errors in the timing of surgery.

In conclusion, we reviewed our 26-year experience of 144 patients aged 60 years or older who underwent surgery for UC. Our results reinforce that emergency surgery is a predictor of adverse outcome for elderly patients, whereas low complication and death rates should be expected for elective UC surgery in elderly patients.
